# The Quality and Functional Improvement of Retorted Korean Ginseng Chicken Soup (Samgyetang) by Enzymolysis Pre-Treatment with *Cordyceps militaris* Mushroom Extract

**DOI:** 10.3390/foods11030422

**Published:** 2022-01-31

**Authors:** Farouq Heidar Barido, Sun Moon Kang, Sung Ki Lee

**Affiliations:** 1 Department of Applied Animal Science, College of Animal Life Sciences, Kangwon National University, Chuncheon 24341, Korea; farouq.heidar12@gmail.com; 2 National Institute of Animal Science, Rural Development Administration, Wanju 55365, Korea; smkang77@korea.kr

**Keywords:** enzymolysis, protein hydrolysis, functional improvement, meat quality, chicken soup, *Cordyceps militaris* mushroom

## Abstract

This study aimed to investigate the functional and quality improvement of retorted Korean ginseng chicken soup that was hydrolyzed using a single extract from *Cordyceps militaris* (CM) mushroom, or in combination with bromelain, flavorzyme, or a mix of both. A total of 36 fat-trimmed breast meat from commercial broilers were hydrolyzed with one of six treatments, (1) flavorzyme as a positive control (PC), (2) no addition as negative control (NC), (3) crude CM extract (CME), CM extract prepared with either (4) bromelain (CMB), (5) flavorzyme (CMF), or (6) bromelain:flavorzyme mixture (CMBF) in a water bath at 55 °C for 2.5 h, and subsequently retorted at 121.1 °C, 147.1 kPa for 1 h. The highest antioxidant activity was observed in the CMB treatment (40.32%), followed by CMBF (34.20%), and CME (32.97%). The suppression of malondialdehyde ranged between 28 and 83%. The water-holding-capacity of the treated samples increased, ranging between 59.69 and 62.98%, and significantly tenderized the meat. The shear force decreased from 23.05 N in negative control to 11.67 N in the CMB samples. The predominant nucleotides across the samples were 5′-IMP and hypoxanthine, and the lowest was adenosine. The intensification of the taste properties was due to the increase of umami substances, both by 5′-nucleotides (5′-IMP, 5′-GMP) and free amino acids (FAAs), whereas the highest improvement was observed in the CMB group. Therefore, the hydrolyzation of Korean ginseng chicken soup using CM extract, prepared using bromelain, improves functional and quality profiles.

## 1. Introduction

Consumer motivation toward food consumption has been steadily shifting from hunger satisfaction and basic nutritional fulfillment to an expectation of health-promoting and disease-prevention effects [1]. Although the three main quality indices of food acceptance; flavor, nutrition, and mouth feel, are still major determinants of consumer repurchasing preference, countless studies in the last 10 years have been carried out to develop food products with health benefits [2]. Chicken soup is one of the most consumed food cuisine with abundant nutritional value. It is enriched with high protein content, free amino acids (FAAs), polyunsaturated fatty acids (PUFAs), and polysaccharides, which makes it ideal for health maintenance [3]. In Korea, a ginseng chicken soup or Samgyetang is a traditional chicken soup that is made with the addition of ginseng that is popular during summer season to replace nutrient loss because of the excessive activities and hot temperatures. Nowadays, the food industries manage to provide Samgyetang in ready-to-eat pouch to ease busy households [4]. Wherein, retort processing is generally used by the food industries to prepare for ready-to-eat Samgyetang. Retorting is one of the highly applied methods for manufacturing shelf-stable products. It employs extremely high temperatures and pressures at determined periods of time, thus eliminates pathogenic microorganisms [5,6].

Studies have shown that meat proteins exert numerous effects, including pharmaceutical effects against cardiovascular disease (CVD) that is caused by high blood pressure via the inhibition of angiotensin-I converting enzyme (ACE) [7,8]. Chicken meat was reported to have ACE-inhibiting peptides with IC_50_ values of approximately 3.2–14 µM [9]. However, these functions depend on the digestibility of the proteins in the small intestine, suggesting that free peptides exert a greater effect following consumption [10]. Hence, the chemical or physical hydrolyzation of meat protein to generate small chain peptides has been studied extensively, with enzymatic hydrolysis emerging as a suitable method for producing controlled end-products without forming toxic catabolites and carcinogens [11,12].

Apart from its notable function in the production of highly functional peptides, enzymolysis pre-treatment of chicken meat has been shown to enhance the flavor profile through the release of volatile compounds [13] and the enhancement of protein solubility. However, the selection of appropriate enzymes requires careful investigation. The release of hydrophobic amino acids and the greater exposure of hydrophobic fraction may be a result of the hydrolyzation process, wherein a higher degree of hydrolysis is mentioned to promote the formation of these compounds, thus having a bitter taste. The effort to control the emergance of bitter taste in hydrolysate products can be highly dependent on the peptidase activity, thus careful selection of enzymes should be well performed [14]. A recent study by Polanco-Lugo et al. [14] in a lima bean protein isolate that was obtained via different enzymolysis treatments, showed that the development of umami compounds involving FAAs and 5′-nucleotides was higher with enzyme which had a stronger endopeptidase activity. Nevertheless, Marciniak et al. [15] reported that the endopeptidases, for which the cleaving site is specifically on oligopeptides, may release more hydrophobic amino acids and bitter-containing amino acids, such as Arg, Lys, and Phe. 

The mushroom *Cordyceps militaris* (CM), is pharmacologically known for a myriad of remedies. Abundant bioactive compounds, namely cordycepin, adenosine, and D-mannitol, that are present in CM are believed to have antioxidant, anti-inflammatory, antimicrobial, anti-tumor, and nephroprotective properties [16]. Our previous study on the incorporation effect of dried or fresh CM mushroom on chicken soup demonstrated the possible improvement of chicken meat quality through extensive protection against lipid oxidation, higher antioxidative profiles, and increased non-volatile taste compounds [3]. The upregulation of endogenous proteolytic enzymes due to their high serine and metalloprotease activity during post-mortem tenderization was also discovered after marination of the chicken breast with CM mushroom extract [17]. The moderate proteolytic activity of the CM mushroom at 4.57 unit/mL and abundant 5′-adenosine monophosphate concentration was thought to be responsible for the above-mentioned effects [18]. 

With respect to the possibility of kinetic parameter alteration from heterologous enzyme–enzyme interactions, or the interaction between enzymes with different structures [19], this study proposed the application of pre-enzymatic treatment using CM mushroom extracts on chicken breast before being processed through retorting. The taste compounds are expected to be enhanced by enzymatic processes through the increased formation of free amino acids and short chain peptides. In addition, the functional properties from the incorporation of the CM mushroom extract into Samgyetang were expected. By comparing the single CM mushroom extract effect with that of CM mushroom extract that previously reacted with the enzymes, the results of this study could provide pivotal information on the efficacy of the hydrolysis pre-treatement by using suitable CM mushroom extracts for the improvement of functional and taste properties of the Korean ginseng chicken soup.

## 2. Materials and Methods

### 2.1. Preparation of Cordyceps Militaris Extract

The CM mushroom extracts were prepared according to the methods by Barido and Lee [17] and Sukkhown et al. [20] with minor modification. Briefly, finely-ground fresh CM mushroom fruiting bodies were mixed with water at (1:2, *w*/*v*) using a food blender (EBR9814W, Electrolux, Stockholm, Sweden) at 13,500× *g* for 1 min, and placed at 4 ± 2 °C for 24 h to generate a crude CM mushroom extract (CME). With regard to the possible enzyme–enzyme interactions, the mushroom slurries were added with three proteolytic enzyme mixtures, each having different peptidase activities; bromelain (CMB), flavorzyme (CMF), and mixture of bromelain: flavorzyme (CMBF) at (1:1), and then incubated in a water bath (BW-20G, Biotechnical Services, North Little Rock, AR, USA) at 55 °C for 20 h [11]. The pH adjustment was set at 6.5 before the incorporation of enzymes, and the addition amount were set at 1% of the dry matter base [21]. The mixture solutions were then centrifuged (1248R, Labogene, Lynge, Denmark) at 1370× *g*, 4 °C for 30 min, filtered with Whatman filter paper no. 1.

### 2.2. Preparation of the Korean Chicken Soup

A total of 72 skinless commercial broiler (breast) muscle (300 ± 15 g) were purchased from a local commercial slaughterhouse. The visible fat was removed inside the cooling room (4 ± 2 °C) and the samples were dried with a paper towel. The incorporation percentage of the CM mushroom extract was determined based on the result from pre-experimental studies, wherein the concentration of CM mushroom extract at (0%, 2%, 3%, and 5%) were compared. The fix percentage of addition was optimized using shear force value, protein solubility, lipid oxidation rate, and taste-related compounds data by a single factor experiment. Based on the results, the effect of CM mushroom extract on chicken soup was likely dose-dependant, wherein the most improved effect was observed at more than 3% addition (data not shown), and 5% addition (*v*/*w*) was selected as the fixed amount. Subsequently, the breast meat was hydrolyzed with one of six treatments, (1) flavorzyme as positive control (PC), (2) no addition as negative control (NC), (3) crude CM extract (CME), and CM extract that reacted with either (4) bromelain (CMB), (5) flavorzyme (CMF), or (6) bromelain: flavorzyme mixture (CMBF) in a water bath at 55 °C for 2.5 h, according the method of Kong et al. [22]. Following hydrolyzation, the prepared broth (300 mL) containing 0.6% salt was added based on the method by Jeong et al. [23], and processed through retorting with retort machine at 121.1 °C, 147.1 kPa for 1 h. Each treatment group had 6 replications. After the process was finished, the breast samples were separated from the broth for further analyses. As each pouch was composed of two breasts, one breast was subjected to physicochemical analysis and remaining breast was for the texture properties. Meanwhile, the broth samples were collected from each sample through filtration using a mesh filter, centrifuged at 3500 rpm at 15 °C for 30 min, and stored at −20 °C until analysis. The analyses on the broth sample were performed to measure the effect of hyrdrolyzation on the taste-related compounds and free amino acid contents. The quality and functional properties, including proximate composition, pH value, surface color, shear force, water-holding capacity, cooking loss, taste-related nucleotides, free amino acid contents, and antioxidative properties, were measured on the breast sample.

### 2.3. Proteolytic Activity

The proteolytic activity of the extracts were measured as the proteolytic activity and was determined by using casein as the substrate. One unit of caseinolytic activity was defined as the amount of enzyme that caused an increase of 0.1 absorbance units at 280 nm after 60 min incubation (MIR-153, Sanyo Electric Co., Ltd., Osaka, Japan) at 35 °C.

### 2.4. Proximate and pH Value

The proximate analyses were conducted following a method by Association of official analytical collaboration (AOAC) [24]. The moisture percentage was obtained after drying 1 g of the breast at 105 °C for 24 h. The crude protein percentage was determined through Kjeltec system procedure (2200 Kjeltec Auto Distillation Unit, Foss, Hillerød, Denmark). The crude fat was determined by the Soxhlet extraction method for 48 h, while the crude ash content was obtained after burning in a muffle furnace (LEF-115S, Daihan Labtech Co., Ltd., Namyangju, Korea) at 550 °C. All analyses were performed in triplicate. The pH value was tested on cooked breast samples in triplicate. After mixing 5 g of the sample with 45 mL of distilled water in a homogenizer (PH91; SMT Co., Ltd., Tokyo, Japan), the pH of the stirred slurry samples was measured using a pH meter probe (Seven Easy pH; Mettler-Toledo GmbH, Schwerzenbach, Switzerland) that had previously been calibrated.

### 2.5. Surface Color

The visual appraisal of the breast meat samples were done by the colorimeter measurement (Chromameter CR-400, Konica Minolta Sensing, Osaka, Japan) on 5 different points per sample. The chromameter calibration was previously calibrated by using a white plate (2° observer; Illuminant C: Y = 93.6, x = 0.3134, y = 0.3194).

### 2.6. Shear Force Value, Water-Holding Capacity (WHC), and Cooking Loss

The shear force value was performed to evaluate the profile of meat tenderness after treatments. Its value was determined based on 1.5 × 1.5 × 1.5 cm^3^ samples that were prepared in triplicate under the V blade and cut with constant speed parameters using a TA-XT2i Plus (Stable Micro Systems, Surrey, UK). The water holding capacity (WHC) percentage was obtained following the protocol of Kristensen and Purslow [25] by the centrifugation method. The WHC percentage was obtained by calculating the ratio of the total moisture content to the remaining water volume after being subjected to boiling in a water bath followed by centrifugation. Meanwhile, the cooking loss percentages of the samples were obtained by calculating the weight before and after cooking ((W1−W2)−W1).

### 2.7. Antioxidative Properties

The antioxidant activities of the breast samples were determined using DPPH assays according to the method by Islam et al. [26] in triplicate. The results were expressed as a scavenging percentage of the free radicals. In addition, the 2-thiobarbituric acid reactive substances (TBARS) method was used to quantify the malondialdehyde concentration; secondary metabolites from the lipid oxidation. Briefly, the chicken breast samples (0.5 g) were prepared in triplicate in a 25 mL TBARS test tube, adding an antioxidant mixture (0.1 g). A total of 3 mL of 1% TBA in 0.3% NaOH was added to the mixture, which was then vortexed, followed by the addition of 17 mL of 2.5% trichloroacetic acid in 36 mM HCl. After the tubes were sealed, they were heated in a water bath (BW-20G, Biotechnical Services, North Little Rock, AR, USA) at 100 °C for 30 min. The tubes were directly immersed in ice water once the heating was completed. A total of 5 mL of aqueous sample was moved to a new 15 mL conical tube and mixed with 3 mL of chloroform. The mixtures were subsequently subjected to centrifugation at 2400× *g* for 30 min at 4 °C (1248R, Labogene, Lynge, Denmark). The absorbance was recorded at 532 nm using a UV spectrophotometer (UV-mini 1240 PC, Shimadzu, Kyoto, Japan) and compared against a blank.

### 2.8. Taste-Related Nucleotides

Quantification of the 5′-nucleotides, including adenosine monophosphate (AMP), inosine monophosphate (IMP), guanosine monophosphate (GMP), adenosine, and hypoxanthine, was performed using HPLC (HPLC; Nexera X2 HPLC, Shimadzu, Kyoto, Japan) according to a previous study by Jayasena et al. [27]. The mobile phase consisted of A 0.04% (*v*/*v*) triethylamine in phosphate buffer (58.72 mM Na_2_HPO_4_, 40 mM KH_2_PO_4_, pH 7.02 at 22 °C), and B was a mixture of HPLC-grade distilled water and acetonitrile (40:60, *v*/*v*). The analysis was conducted in triplicate, wherein the absorbance was measured at 260 nm using diode array detectors, and the identified peaks were compared with the retention time of the prepared standards (Sigma-Aldrich, St. Louis, MO, USA).

### 2.9. Free Amino Acid Contents

The FAA was determined in triplicate according to the method of Rahman et al. [28] with slight modifications. Finely ground sample (500 mg) and 6 N HCl (20 mL) were placed into a 25 mL test tube. After being flushed with N_2_ gas for 30 s, a sealed tube was subjected to hydrolysis at 110 °C for 16 h. Then, 100 μL of the hydrolyzed solution was evaporated under N_2_ gas for another 30 s, dissolved with 1 mL of Milli-Q water, vortexed, and the mixtures were filtered through a 0.45 μm PTFE filter. The FAA was quantified by high-performance liquid chromatography (HPLC; Nexera X2 HPLC, Shimadzu, Kyoto, Japan) after derivatization with opthaldialdehyde 2 min prior to injection. The column size was 4.6 × 150 mm, with a 5 μm particle size, (Agilent Technologies), and a 338 nm detection wavelength at 40 °C. Mobile phase A was 40 mM NaH_2_PO_4_, pH 7.8, and mobile phase B was 45% acetonitrile, 45% methanol, and 10% Milli-Q water, with the separation performed at a 1.5 mL/min flow rate. The details of the gradient program were: 2% B for 0 and 0.5 min; 57% B for 20 min; 100% B for 20.1 and 23.5 min; 2% and 0% B for 23.6 and 25 min respectively; the stop-time and post-time were 25 and 5 min, respectively.

### 2.10. Statistical Analysis

The data analyses in this study were performed using one-way analysis of variance (ANOVA) using R version 3.6.1 (The R-foundation for Statistical Computing, Vienna, Austria). The significant mean value of each group following the treatment on proteolytic acitivity, proximate composition, pH value, surface color, shear force, water-holding capacity, cooking loss, taste-related nucleotides, free amino acid contents, and antioxidative properties were continuously analyzed using Duncan’s multiple range test, with statistical significance set at *p* < 0.05. The results are presented as average values and the standard error of the mean (SEM).

## 3. Results

### 3.1. Proteolytic Activity

The proteolytic activity of the CM mushroom extracts are shown in Table 1. A solution containing CMB protease accounted for 4.57 unit/mL, indicating the highest proteolytic activity among the CM protease groups (*p* < 0.05). Single proteolytic activity from the CM mushroom extract, as seen in CME, was observed the lowest with 2.90 unit/mL, below the CMF (3.89 unit/mL) and CMBF (4.01 unit/mL). The results implied that there might be improvement on the proteolytic activity of the CM mushroom extract after reaction with various enzymes during hydrolysis. The effort to optimize the protease performance may vary. Simple temperature and pH treatment were enough to modify the structural properties of the protease and lead to subsequent functional alterations [29]. The optimization may include the inhibition of autodigestion of protein and enzyme–enzyme reaction that permits the cleavage of the protein into certain amino acids which have potent proteolytic activity [19]. Besides, the serine protease from CM mushroom may exhibit a mild proteolytic activity toward some substrate, including myfobrillar protein with limitation on sarcoplasmic protein and connective tissue [30]. The mild protease has often been proven to impart a positive tenderization effect on meat, due to a controllable end product. Unlike papain which has a broader substrate and high proteolytic activity and which are mentioned to possibly hydrolize both connective tissue and myofibrillar proteins, thus promote to unexpected side-effects [31].

### 3.2. Proximate and pH Value

The negative control that was used in this study contained 68.16% moisture, 27.69% (86.97, dry basis) crude protein, 2.35% (7.38, dry basis) crude fat, and 1.10% (3.45, dry basis) ash content. Following treatment with various extracts, the moisture and crude protein percentages were significantly altered where both the positive control and the treatment groups had a significantly higher moisture percentage compared to the negative control (*p* < 0.05). As shown in Table 2, the protein content decreased significantly following treatment with extract in all the sample groups. Similarly, the positive control, in which flavorzyme was used as a hydrolyzation agent, had a significantly lower protein content (26.17%) than the negative control. Furthermore, the crude fat and ash percentages did not differ among the samples (*p* > 0.05). The increase in moisture percentage may be attributed to the hydrophilicity alteration of the meat proteins following enzymolysis [32], causing more interaction between the free peptides and water molecules [20,33]. This is in accordance with the previous studies by [32,34,35] who found an increase in the moisture percentage after incorporating proteolytic enzyme-rich plant extract. However, the enzymolysis process can also cause degradation of myofibril protein and connective tissue, resulting in a lower protein content [36], as observed in the present study.

pH is an important initial quality determinant of meat quality [37], and this study observed a significant increase in pH after enzymolysis pre-treatment with various extracts. The highest increment was observed in the sample groups that were treated with the CMB and CMF ranging between pH 6.75 and 6.79. The samples that were hydrolyzed with CMBF had a similar pH value to the positive control (*p* > 0.05), and significantly higher than the negative control (pH 6.51) and the CME-treated groups (pH 6.57). The main consequence of enzymolysis was the cleavage of intact proteins into peptides with lower molecular weights. In contrast to proteins, free peptides with lower molecular weights have a stronger binding ability to immobilize water [38], and subsequently influence the percentage of retained water after processing. The results of this study are in agreement with those of [16,35,39].

### 3.3. Surface Color

The instrumental color of the meat of the negative control was used as a reference for color differences. The lightness (L*), redness (a*), and yellowness (b*) values in the negative control were recorded as 72.13, 3.24, and 20.14, respectively. As presented in Table 3, enzymolysis pre-treatment produced meat with a lighter color profile regardless of the utilized extract compared to the negative control (*p* < 0.05). The lightest surface color was observed for chicken meat that was treated with either CMB or CMF. In addition, the flavorzyme-treated group, either alone (PC) or in combination with the CM extract (CMF) significantly intensified the redness and had the highest redness value among the treatments. A similar pattern was also observed for yellowness. The results of this study were in accordance with those of Gao et al. [11] and Ang et al. [21], wherein protein hydrolysis resulted in a lighter color. The reason might be due to the separation of globin from the heme group as a result of protein hydrolysis that inhibits the formation of dark color, thus resulting in the lighter color of the hydrolysate [40]. 

### 3.4. Shear Force Value, Water-Holding Capacity (WHC), and Cooking Loss

The textural properties of the chicken meat samples that were measured by the Warner-Bratzler shear force are presented in Table 4. The shear force value decreased dramatically from 23.05 N in the negative control to the lowest at 11.67 N in the CMB-hydrolyzed samples (*p* < 0.05). The textural properties of the positive control differed significantly from those of the negative control, but did not differ from the other treatments, indicating the efficacy of enzymolysis pre-treatment in softening the meat texture (Table 4). Accordingly, the WHC of the treated samples showed a significant increase (ranging between 59.69–62.98%) compared to that of the negative control (56.22%). However, this study revealed no differences in WHC among the different extract treatments (*p* > 0.05). After undergoing processing, along with the flavor, the texture properties of the meat hold essential role to determine the consumer repurchasing intention, thus it has become one of the highly studied objects in the last decades [41]. Previous studies have suggested a strong correlation between a high WHC and the occurrence of protein solubility [12,42,43], which, in turn, exposes more binding sites for water [44]. The enzymolysis pre-treatment was proven to increase the protein solubility rate through the degradation of intact proteins into small chain proteins and peptides [45]. In addition, the higher tenderization activity of the CM extract that was prepared with bromelain, might be due to the synergistic effect between the endopeptidase activity of bromelain to facilitate more protein release and degradation via protein internal chain cleavage [19]. The textural improvement in meat after hydrolyzation is thought to be the result from the increased protein solubility of the small peptides and the consistent increase of the carboxyl group and ionizable amino acids [45]. Different peptidases contribute differently towards the functional properties improvement. Alcalase enzyme has a potent endopeptidase that was mentioned to contribute for texture improvement through the decrease of surface hydrophobicity [46,47]. Similarly, the flavorzymes which are strong in exopeptidase, have a broader range of substrate specifity, thus contibute to texture improvement. However, the small peptides interaction may be observed on flavorzyme-hydrolyzed products, causing self-petide aggregation and the increased exposure of hydrophobic peptides that eventually increase the protein insolubility [45]. As also seen as a result of this study, the endopeptidase from bromelain may create a synergistic interaction with the serine extract from CM mushrooms and establish the more effective extract as a meat tenderizer. Our previous study revealed that the endogenous enzyme that was related to tenderization was upregulated by the CM extract, leading to higher myofibril protein fragmentation and resulting in a more tender texture of spent hen breast meat [17]. This agrees with the study by Choi et al. [30], who mentioned the possibility of purified CM enzyme binding- and cleaving-specific sites in myofibril protein. No marked differences were found in cooking loss percentages among the samples.

### 3.5. Antioxidative Properties

**Figure 1:**
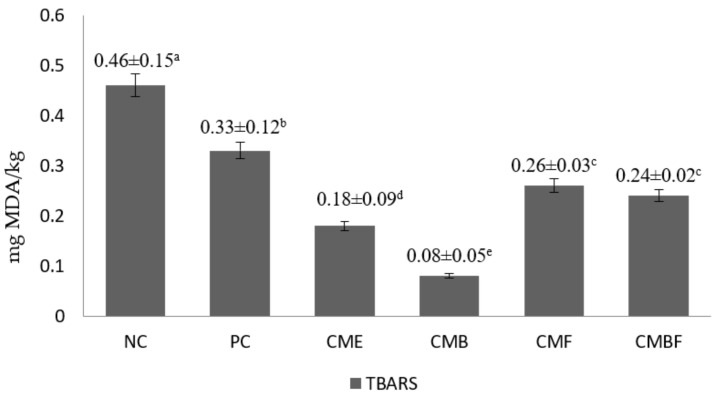
The lipid oxidation rate of the breast meat of Korean ginseng chicken soup that was hydrolyzed with *Cordyceps militaris* mushroom extract. NC, negative control; PC, Positive control, Samgyetang that was hydrolyzed with flavorzyme before retorting; CME, Samgyetang that was made with the addition of *Cordyceps militaris* mushroom extract; CMB, Samgyetang that was hydrolyzed with *Cordyceps militaris* mushroom extract: bromelain before retorting; CMF, Samgyetang that was hydrolyzed with *Cordyceps militaris* mushroom extract: flavorzyme before retorting; CMBF, Samgyetang that was hydrolyzed with *Cordyceps militaris* mushroom extract: bromelain:flavorzyme before retorting. The values with different superscripts were significantly different (*p* < 0.05).

**Figure 2:**
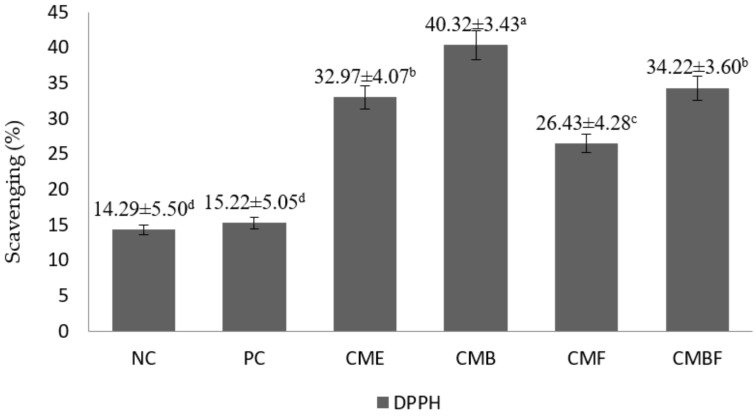
The antioxidative profile of the breast meat of Korean ginseng chicken soup that was hydrolyzed with *Cordyceps militaris* mushroom extract. NC, negative control; PC, Positive control, Samgyetang that was hydrolyzed with flavorzyme before retorting; CME, Samgyetang that was made with the addition of *Cordyceps militaris* mushroom extract; CMB, Samgyetang that was hydrolyzed with *Cordyceps militaris* mushroom extract: bromelain before retorting; CMF, Samgyetang that was hydrolyzed with *Cordyceps militaris* mushroom extract: flavorzyme before retorting; CMBF, Samgyetang that was hydrolyzed with *Cordyceps militaris* mushroom extract: bromelain:flavorzyme before retorting. The values with different superscripts were significantly different (*p* < 0.05).

The antioxidative potential of the chicken breast meat samples that were hydrolyzed with various CM mushroom extracts was measured by DPPH scavenging activity and TBARS assay (Figure 1 and Figure 2). The DPPH radical scavenging activity percentage of the negative control was 14.29%. It was not significantly different from that of the positive control at 15.22% (*p* > 0.05). Furthermore, the radical scavenging activity was observed to be the highest in the meat samples that were hydrolyzed with CMB (40.32%), followed by CMBF (34.2%), CME (32.97%), and CMF (26.43%), respectively. Interestingly, the CME-treated samples showed significantly higher antioxidative activity in comparison to the CMF-treated samples (*p* < 0.05). In addition, when confirmed by the TBARS assay, the suppression of malondialdehyde (MDA) as the secondary metabolite from lipid oxidation ranged between 28 and 83% when compared to the negative control, with the strongest reduction that was observed in the sample group that was hydrolyzed by CMB (*p* < 0.05). Subsequently, MDA-suppressing activity from highest to lowest in this study was CME, CMBF, CMF, PC, and NC, respectively. The enzymolysis pre-treatment groups shared similar lipid oxidation-suppressing activities and were not significantly different (*p* > 0.05), except for the positive control when compared to CME (*p* < 0.05). The CME-treated group had a markedly lower MDA content (0.18 mg MDA/kg) when compared to the positive control (0.33 mg MDA/kg). 

The results of this study confirmed our previous report that the incorporation of CM mushroom extract into Samgyetang resulted in improved antioxidant activity in a dose-dependent manner [3]. The CM mushroom extract alone may exhibit a potent scavenging activity toward stable free radical DPPH. The scavenging activity that is possessed by CM mushroom extract ranged from 30.12 to 46.96% [3]. The polysaccharides along with the abundant bioactive compounds, especially cordycepin, adenosine, and phenolic acids, are believed to exert pharmacological effects, including antioxidant activity [48]. Consequently, the single hydrolyzation by CM mushroom extract demonstrated an increase in the antioxidative profile of the chicken soup in comparison with the negative control. In addition, antioxidant compounds have been widely recognized to play a significant role in the inhibition of lipid oxidation [49]. The antioxidant activity of chicken meat is influenced by numerous factors, including amino acids, histidine-containing peptides, uric acid, and polyamines. However, the antioxidant activity of these peptides is poor within intact proteins. Thus, hydrolysis aims at increasing the cleavage of intact proteins, resulting in more free peptides [50]. Furthermore, different peptidases are of crucial importance as they rule end-product functionality, wherein endopeptidase-hydrolyzed products are generally enriched with more antioxidant amino acids when compared to exopeptidase-hydrolyzed products [11,20,22]. Thus, this study agrees with previous reports, as endopeptidases in bromelain combined with CM extract gave higher antioxidative activity than exopeptidases in flavorzyme [11].

### 3.6. Taste-Related Nucleotides

The taste-related nucleotides in both breast and broth samples were analyzed and are presented in Table 5. The predominant nucleotides that were observed across the samples were 5′-IMP and hypoxanthine, while the lowest was adenosine. There are two main nucleotide compounds that may impart the umami taste in meat; the purine ribonucleotides with a phosphate ester on the 5′-carbon of the ribose section and a hydroxyl group on the 6′-carbon of the purine ring. Meanwhile, the strength of umami properties given by these nucleotide compounds are depend on its structure. The 5′-GMP, 5′-IMP, and 5′-AMP are mentioned as potent umami compounds, while the purine nucleotide with 6′-carbon at the purine section, such as inosine and adenosine may also hold the umami taste, but at a weaker level than that of the ribonucleotide with phosphate ester at the C-5 of the ribose section [51]. In addition, the purine ribonucleotides that are phosphorylated at C-2′ or C-3′ of the ribose section can be classified as tasteless, or a potential umami compund when they are reacted with certain amino acids [52]. 

The results of this study showed that the concentration of umami-related nucleotides in breast meat samples increased remarkably following enzymolysis pre-treatment, regardless of the extract that was used. Whereas the highest concentration of 5′-IMP in this study were observed for CMB (51.78 µg/mL), followed by PC (47.80 µg/mL), CME (47.34 µg/mL), CMF (44.81 µg/mL), CMBF (43.65 µg/mL), and NC (16.23 µg/mL), respectively. Similarly, the 5′-GMP concentration was the highest in the CMB-treated group at 21.36 µg/mL (*p* < 0.05). However, this study found no significant difference in 5′-GMP concentration between PC (12.22 µg/mL) and NC (12.20 µg/mL). Meanwhile, the umami-related nucleotide contents in the broth samples showed the highest result for the CMBF-treated groups. The 5′-GMP, 5′-IMP, and 5′-AMP concentration in broth of CMBF was observed at 26.31 µg/mL, 38.24 µg/mL, and 22.23 µg/mL, respectively. Whereas, the lowest umami-related nucleotide in the broth samples was observed in the negative control, wherein the 5′-GMP, 5′-IMP, and 5′-AMP concentration was at 5.45 µg/mL, 13.39 µg/mL, and 5.12 µg/mL, respectively. Based on the results, the enzymolysis pre-treated samples had significantly higher umami-related nucleotides when compared to the negative control. The results of this study confirmed the findings of previous reports [21,32,39,52], that demonstrated the benefits of meat protein hydrolyzation as a taste enhancer. The combination of endopeptidases and exopeptidases in CMBF may utilize broader substrate specificity by using both amino- and carboxyl-terminal internal peptide chains as cleavage sites [11,21,22].

### 3.7. Free Amino Acid Contents

Table 6 and Table 7 display the FAA contents following enzymolysis pre-treatment with various CM mushroom extracts. A total of 16 FAAs were identified in this study via HPLC analysis. These FAAs are highly responsible for the taste properties and development of flavour-forming reaction of meat. Each amino acid may contribute to the taste differently [36]. Umami taste is related to Asp and Glu; sweet taste to Ser, Gly, Thr, Ala, and Pro; and bitter taste to Val, Ile, Met, Lys, Leu, Phe, Arg, His, and Tyr [53]. The total amount of identified FAAs in the breast meat of the negative control was 1680.97 µg/mL. This was lower than that which was previously reported by Qi et al. [54] in yellow-feathered chicken (2585.20 µg/mL), and by Rikimaru et al. [55] in Japanese yellow-feathered chicken meat. In addition, its total concentration was significantly higher in the positive control, both in breast meat and broth samples (*p* < 0.05). Concerning the different taste-sensations, individual bitter amino acids (His) were the predominant FAAs, ranging between 474.65 and 508.93 µg/mL in the chicken breast, and 456.85 and 466.04 µg/mL in the broth. The umami-related amino acids (Asp, Glu) in the cooked breast were found the highest in PC (368.42 µg/mL), followed by CMBF (344.86 µg/mL), CMF (342.62 µg/mL), CMB (328.43 µg/mL), CME (312.20 µg/mL), and NC (298.67 µg/mL), respectively (*p* < 0.05). Interestingly, regardless of the used that was extract, the production of the umami substances was apparently higher compared to the direct incorporation of the CM mushroom. However, with respect to the broth sample, the CMB-treated groups produced the highest umami amino acids (Asp, Glu) among the samples (335.76 µg/mL), unless from the CMBF (328.42 µg/mL). The total umami amino acids in PC, CME, and CMF were (324.20, 326.90, and 325.81 µg/mL), respectively. In addition, all the treatments showed significantly higher umami amino acids in comparison with that of NC (212.89 µg/mL), and suggested a potential implication of the extract from CM mushroom as a taste enhancer. Regarding to the sweet taste, the highest total sweet amino acids (Ala, Gly, Thr, Ser, Pro) in the breast samples were observed for PC (683.97 µg/mL), followed by CMBF (619.40 µg/mL), CMB (619.36 µg/mL), CMF (613.80 µg/mL), CME (587.75 µg/mL), and NC (549.49 µg/mL), respectively. Whereas, the highest sweet amino acids in the broth samples were recorded for the CMB-treated group (602.32 µg/mL), followed by CMBF (583.97 µg/mL), CME (580.17 µg/mL), CMF (577.37 µg/mL), PC (573.42 µg/mL), and NC (335.14 µg/mL), respectively. The results of this study revealed that hydrolysis pre-treatment by using CM mushroom extracts at any condition significantly intensified the sweet sensation of both the breast and broth samples when compared with that of group that received no hydrolyzation. Meanwhile, considering the bitter amino acids (Val, Ile, Met, Leu, Phe), the CMB-treated group generated the predominant bitter sensation in both the breast and broth samples with 355.31 and 193.26 µg/mL, respectively. Furthermore, with respect to the bitter amino acids in the breast sample from the highest to the lowest was CMBF (295.48 µg/mL), CMF (289.88 µg/mL), NC (275.35 µg/mL), PC (274.88 µg/mL), and CME (263.83 µg/mL), respectively. Similarly, the bitter amino acids in the broth samples were at 174.88, 171.08, 168.28, 164.33, and 84.53 µg/mL for CMBF, CME, CMF, PC, and NC, respectively. The more exposure of the hydrophobic fraction may be the result from the hydrolyzation process with endopeptidase enzyme [22]. It was also revealed by this study that using bromelain as an enzyme to react with mushroom extracts may have strong antioxidant compounds and they might also impart bitter sensation. Furthermore, concerning the acid taste in the breast sample after hydrolysis, the PC-treated group had the highest total acid taste amino acids (Asp, Glu, His) with 847.78 µg/mL, followed by CMB (837.74 µg/mL), CMBF (825.84 µg/mL), CMF (822.48 µg/mL), NC (807.60 µg/mL), and CME (786.85 µg/mL), respectively. Moreover, the order of the broth samples with acid taste concentrations from the highest to the lowest was CMB (798.83 µg/mL), CMBF (787.38 µg/mL), CME (785.10 µg/mL), CMF (783.45), PC (781.05 µg/mL), and NC (678.93 µg/mL).

Furthermore, regardless of the extract that was used, the total FAAs concentration significantly increased following enzymolysis pre-treatment in either the breast meat or the broth samples (*p* < 0.05). The intensification of FAAs in the enzymolysis pre-treated samples may be attributed to the proteolytic nature of the cleaving and breaking down of intact proteins into shorter soluble peptides. In addition, the strong synergistic activity of both endopeptidases and exopeptidases in CMBF may have produced a more intense umami taste in the flavorzyme and the bromelain-treated chicken soup [11,13]. Apart from having plentiful bioactive compounds, many studies also unraveled the taste-related compounds that are contained within the CM mushroom. The greatest contributor for taste-related compounds from the CM mushroom are believed to be from the 5′-AMP, soluble sugars, free amino acids, and volatile compounds [56]. In addition, the abundant monosodium glutamate-like amino acid in the CM mushroom [57] may have also contributed to the umami taste, as was also found in our previous study.

## 4. Conclusions

The potential application of either single CM mushroom extract, or CM mushroom extract that is prepared with various enzymes as functional and taste enhancers of the retorted Korean ginseng chicken soup was carefully investigated. The potential serine protease from CM mushroom was prepared with diverse enzymes which had different peptidase activities with respect to kinetics alteration from the enzyme–enzyme interaction. The utilization of different enzymes that were added in the CM mushroom extract contributed differently to the development of both functional and taste properties of the retorted Korean ginseng chicken soup. The highest antioxidant activity for scavenging free radicals was observed in the meat that was hydrolyzed with CMB (40.32%), followed by CMBF (34.2%), CME (32.97%), CMF (26.43%), PC, and NC, respectively. Additionally, the suppression of MDA ranged between 28 and 83% compared to the negative control. The WHC of the treated samples increased, ranging between 59.69 and 62.98%, and contributed to significant tenderization of the meat texture. The shear force value decreased dramatically from 2.35 kgf in the negative control to the lowest at 1.19 kgf in the CMB-hydrolyzed samples. The predominant nucleotides that were observed across the samples were 5′-IMP and hypoxanthine, and the lowest was adenosine. The intensification of umami substances, both by an increase of 5′-nucleotides (5′-IMP, 5′-GMP) and FAAs was observed in this study, with the highest improvement observed in the CMB- and CMBF-treated samples. This study demonstrated the potential application of CM mushroom extract that is prepared with bromelain to hydrolyze chicken meat before retorting could improve both the functionality and meat quality of Korean ginseng chicken soup.

## Figures and Tables

**Table 1:** Proteolytic activity of the *Cordyceps militaris* mushroom extract.

Variables	Treatments ^1^					SEM	*p*-Value
	PC	CME	CMB	CMF	CMBF		
Enzyme activity (unit/mL)	1.57 ± 0.75 ^e^	2.90 ± 0.68 ^d^	4.57 ± 0.29 ^a^	3.89 ± 0.61 ^c^	4.01 ± 0.15 ^b^	0.09	<0.05

^1^ PC, Positive control, flavorzyme; CME, *Cordyceps militaris* mushroom extract; CMB, *Cordyceps militaris* mushroom extract that was hydrolyzed with bromelain; CMF, *Cordyceps militaris* mushroom extract that was hydrolyzed with flavorzyme; CMBF, *Cordyceps militaris* mushroom extract that was hydrolyzed with bromelain: flavorzyme mixture. SEM, the standard error of the mean. ^a–e^ The mean values within the same row with different superscripts are significantly different among the treatments (*p* < 0.05).

**Table 2:** The proximate composition of the breast meat of Korean ginseng chicken soup that was hydrolyzed with *Cordyceps militaris* mushroom extract.

Variables	Treatments ^1^						SEM	*p*-Value
	NC	PC	CME	CMB	CMF	CMBF		
Moisture (%)	68.16 ± 0.02 ^b^	69.61 ± 0.05 ^a^	69.82 ± 0.09 ^a^	70.62 ± 0.11 ^a^	71.32 ± 0.04 ^a^	70.86 ± 0.01 ^a^	0.18	<0.05
Crude fat (%)	2.35 ± 0.01	2.51 ± 0.02	2.34 ± 0.03	2.19 ± 0.02	2.14 ± 0.03	2.38 ± 0.04	0.08	0.09
Crude protein (%)	27.69 ± 0.15 ^a^	26.17 ± 0.03 ^b^	25.72 ± 0.20 ^b^	26.09 ± 0.17 ^b^	25.44 ± 0.17 ^b^	25.68 ± 0.12 ^b^	0.15	<0.05
Ash (%)	1.10 ± 0.01	1.11 ± 0.02	1.11 ± 0.03	1.10 ± 0.04	1.10 ± 0.01	1.07 ± 0.01	0.00	1.12

^1^ NC, negative control; PC, Positive control, Samgyetang that was hydrolyzed with flavorzyme before retorting; CME, Samgyetang that was hydrolyzed with *Cordyceps militaris* mushroom extract; CMB, Samgyetang that was hydrolyzed with *Cordyceps militaris* mushroom extract: bromelain before retorting; CMF, Samgyetang that was hydrolyzed with *Cordyceps militaris* mushroom extract: flavorzyme before retorting; CMBF, Samgyetang that was hydrolyzed with *Cordyceps militaris* mushroom extract: bromelain:flavorzyme before retorting. SEM, the standard error of the mean. ^a,^^b^ The mean values within the same row with different superscripts are significantly different among treatments (*p* < 0.05).

**Table 3:** The surface color of the breast meat of Korean ginseng chicken soup that was hydrolyzed with *Cordyceps militaris* mushroom extract.

Variables	Treatments ^1^						SEM	*p*-Value
	NC	PC	CME	CMB	CMF	CMBF		
L*	72.13 ± 0.16 ^c^	77.40 ± 0.51 ^a^	74.99 ± 0.43 ^b^	78.71 ± 0.37 ^a^	77.67 ± 0.72 ^a^	73.73 ^b^	0.74	<0.05
a*	3.24 ± 0.09 ^b^	4.26 ± 0.22 ^a^	2.96 ± 0.34 ^b^	3.20 ± 0.39 ^b^	5.38 ± 0.28 ^a^	3.12 ± 0.24 ^b^	0.28	<0.05
b*	20.14 ± 0.71 ^b^	28.85 ± 1.01 ^a^	21.56 ± 1.15 ^b^	17.65 ± 0.98 ^c^	19.13 ± 1.23 ^b,c^	22.80 ± 1.10 ^b^	1.71	<0.05

^1^ NC, negative control; PC, Positive control, Samgyetang that was hydrolyzed with flavorzyme before retorting; CME, Samgyetang that was hydrolyzed with *Cordyceps militaris* mushroom extract; CMB, Samgyetang that was hydrolyzed with *Cordyceps militaris* mushroom extract: bromelain before retorting; CMF, Samgyetang that was hydrolyzed with *Cordyceps militaris* mushroom extract: flavorzyme before retorting; CMBF, Samgyetang that was hydrolyzed with *Cordyceps militaris* mushroom extract: bromelain:flavorzyme before retorting. SEM, the standard error of the mean. ^a–^^c^ the mean values within the same row with different superscripts are significantly different among the treatments (*p* < 0.05).

**Table 4:** The quality characteristics of the breast meat of Korean ginseng chicken soup that was hydrolyzed with *Cordyceps militaris* mushroom extract.

Variables	Treatments ^1^						SEM	*p*-Value
	NC	PC	CME	CMB	CMF	CMBF		
pH	6.51 ± 0.71 ^c^	6.74 ± 0.68 ^b^	6.57 ± 0.47 ^c^	6.75 ± 0.76 ^a^	6.79 ± 0.07 ^a^	6.68 ± 0.04 ^b^	0.02	<0.05
Shear force value (kgf)	2.35 ± 0.07 ^a^	1.39 ± 0.11 ^b^	1.81 ± 0.21 ^b^	1.19 ± 0.09 ^c^	1.26 ± 0.13 ^b,^^c^	1.34 ± 0.19 ^b^	0.10	<0.05
Water holding capacity (%)	56.22 ± 0.35 ^b^	59.69 ± 0.27 ^a^	59.52 ± 0.18 ^a^	61.92 ± 0.25 ^a^	60.73 ± 0.13 ^a^	62.98 ± 0.41 ^a^	0.49	<0.05
Cooking loss (%)	35.34 ± 1.05	35.30 ± 1.21	36.22 ± 0.09	37.21 ± 1.04	36.39 ± 0.07	37.07 ± 0.23	0.64	0.08

^1^ NC, negative control; PC, Positive control, Samgyetang that was hydrolyzed with flavorzyme before retorting; CME, Samgyetang that was hydrolyzed with *Cordyceps militaris* mushroom extract; CMB, Samgyetang that was hydrolyzed with *Cordyceps militaris* mushroom extract: bromelain before retorting; CMF, Samgyetang that was hydrolyzed with *Cordyceps militaris* mushroom extract: flavorzyme before retorting; CMBF, Samgyetang that was hydrolyzed with *Cordyceps militaris* mushroom extract: bromelain:flavorzyme before retorting. SEM, the standard error of the mean. ^a–^^c^ The mean values within the same row with different superscripts are significantly different among the treatments (*p* < 0.05).

**Table 5:** Taste-related nucleotides of the Korean ginseng chicken soup that was hydrolyzed with *Cordyceps militaris* mushroom extract.

	Variables (µg/mL)	Treatments ^1^						SEM	*p*-Value
		NC	PC	CME	CMB	CMF	CMBF		
Breast	5′-AMP	11.56 ± 0.57 ^b^	11.80 ± 0.45 ^b^	12.35 ± 0.72 ^b^	17.560.49 ^a^	13.70 ± 1.03 ^b^	17.69 ± 0.86 ^a^	1.15	<0.05
	5′-IMP	16.23 ± 1.23 ^d^	47.80 ± 2.49 ^b^	47.34 ± 2.76 ^b^	51.78 ± 1.91 ^a^	44.81 ± 0.63 ^c^	43.65 ± 0.52 ^c^	1.51	<0.05
	5′-GMP	12.20 ± 0.21 ^c^	12.22 ± 1.47 ^c^	14.24 ± 2.31 ^b,c^	21.36 ± 1.78 ^a^	16.86 ± 0.96 ^b^	17.68 ± 1.57 ^b^	1.46	<0.05
	Adenosine	0.41 ± 0.07 ^b^	1.76 ± 0.08 ^a^	0.00 ± 0.00 ^c^	0.00 ± 0.00 ^c^	0.00 ± 0.00 ^c^	0.00 ± 0.00 ^c^	0.29	<0.05
	Hypoxhantine	36.54 ± 1.31 ^a^	34.19 ± 2.09 ^a^	29.62 ± 0.08 ^b^	30.62 ± 0.87 ^b^	27.78 ± 0.07 ^c^	27.14 ± 0.11 ^c^	5.27	<0.05
Broth	5′-AMP	5.12 ± 0.11 ^d^	9.03 ± 0.42 ^c^	6.13 ± 0.97 ^d^	13.34 ± 1.37 ^b^	14.33 ± 1.14 ^b^	22.23 ± 1.39 ^a^	2.59	<0.05
	5′-IMP	13.39 ± 0.95 ^c^	23.05 ± 0.74 ^b^	12.66 ± 1.09 ^c^	23.73 ± 0.68 ^b^	25.92 ± 1.97 ^b^	38.24 ± 1.24 ^a^	3.83	<0.05
	5′-GMP	5.45 ± 1.33 ^d^	8.60 ± 2.76 ^c^	6.13 ± 2.54 ^c,d^	16.28 ± 1.17 ^b^	17.18 ± 0.89 ^b^	26.31 ± 0.88 ^a^	3.31	<0.05
	Adenosine	0.86 ± 0.03 ^a^	0.09 ± 0.01 ^b^	0.00 ± 0.00 ^c^	0.00 ± 0.00 ^c^	0.00 ± 0.00 ^c^	0.00 ± 0.00 ^c^	0.14	<0.05
	Hypoxhantine	18.90 ± 3.09 ^e^	33.50 ± 2.89 ^d^	19.07 ± 3.45 ^e^	35.97 ± 0.11 ^c^	41.33 ± 1.04 ^b^	61.96 ± 2.31 ^a^	6.54	<0.05

^1^ NC, negative control; PC, Positive control, Samgyetang that was hydrolyzed with flavorzyme before retorting; CME, Samgyetang that was hydrolyzed with *Cordyceps militaris* mushroom extract; CMB, Samgyetang that was hydrolyzed with *Cordyceps militaris* mushroom extract: bromelain before retorting; CMF, Samgyetang that was hydrolyzed with *Cordyceps militaris* mushroom extract: flavorzyme before retorting; CMBF, Samgyetang that was hydrolyzed with *Cordyceps militaris* mushroom extract: bromelain:flavorzyme before retorting. SEM, the standard error of the mean. ^a–^^e^ The mean values within the same row with different superscripts are significantly different among treatments (*p* < 0.05).

**Table 6:** Free amino acid profiles of the breast meat of Korean ginseng chicken soup that was hydrolyzed with *Cordyceps militaris* mushroom extract.

Variables (µg/mL)	Treatments ^1^						SEM	*p*-Value
	NC	PC	CME	CMB	CMF	CMBF		
Asp	75.20 ± 0.08 ^c^	90.12 ± 0.09 ^a^	82.11 ± 3.14 ^b^	84.21 ± 3.89 ^b^	87.32 ± 5.12 ^a,b^	88.44 ± 3.09 ^a^	5.43	<0.05
Glu	223.47 ± 9.18 ^d^	278.30 ± 0.21 ^a^	230.09 ± 5.66 ^c^	245.22 ± 10.11 ^b^	255.30 ± 6.08 ^b^	256.42 ± 8.74 ^b^	9.86	<0.05
Ser	117.21 ± 0.03 ^b^	115.21 ± 0.05 ^b^	121.77 ± 1.87 ^a^	117.48 ± 0.95 ^b^	126.98 ± 0.87 ^a^	128.10 ± 1.56 ^a^	2.42	<0.05
Gly	85.30 ± 1.37 ^b^	90.22 ± 1.79 ^a^	87.21 ± 2.11 ^b^	85.67 ± 3.77 ^b^	92.42 ± 3.16 ^a^	93.54 ± 2.09 ^a^	3.51	<0.05
Thr	109.11 ± 2.98 ^a,b^	112.31 ± 2.67 ^a^	105.21 ± 1.78 ^c^	108.79 ± 2.02 ^b^	110.42 ± 1.76 ^a^	111.54 ± 2.21 ^a^	2.53	<0.05
Ala	150.21 ± 4.57 ^c^	278.11 ± 3.44 ^a^	188.02 ± 27.65 ^b^	187.21 ± 9.79 ^b^	193.23 ± 26.33 ^b^	194.35 ± 10.87 ^b^	22.31	<0.05
Val	72.29 ± 1.96 ^b^	74.23 ± 2.03 ^b^	60.89 ± 4.09 ^c^	80.11 ± 2.56 ^a^	66.10 ± 4.07 ^c^	67.22 ± 1.68 ^c^	4.80	<0.05
Met	25.47 ± 0.35 ^b^	25.12 ± 0.29 ^b^	25.75 ± 4.11 ^b^	27.84 ± 3.11 ^b^	30.96 ± 2.76 ^a^	32.08 ± 0.08 ^a^	2.00	<0.05
Ile	50.45 ± 0.71 ^c^	49.76 ± 0.65 ^c^	50.55 ± 0.56 ^c^	75.21 ± 5.23 ^a^	55.76 ± 2.30 ^b^	56.88 ± 0.79 ^b^	3.68	<0.05
Phe	48.37 ± 0.04 ^c^	48.28 ± 0.05 ^c^	47.43 ± 0.13 ^c^	79.84 ± 5.05 ^a^	52.64 ± 6.49 ^b^	53.76 ± 3.07 ^b^	5.41	<0.05
Leu	78.77 ± 0.92 ^b^	77.49 ± 0.65 ^b^	79.21 ± 0.88 ^b^	92.31 ± 4.71 ^a^	84.42 ± 6.77 ^a,b^	85.54 ± 0.83 ^a,b^	5.62	<0.05
Arg	90.21 ± 1.87 ^a^	92.32 ± 2.39 ^a^	85.12 ± 2.50 ^b^	89.34 ± 2.37 ^a^	90.33 ± 2.31 ^a^	91.45 ± 1.09 ^a^	2.52	<0.05
His	508.93 ± 3.11 ^a^	478.96 ± 4.75 ^b^	474.65 ± 9.76 ^b^	508.31 ± 8.89 ^a^	479.86 ± 11.71 ^b^	480.98 ± 1.11 ^b^	10.64	<0.05
Tyr	45.98 ± 0.69 ^b^	46.72 ± 0.57 ^b^	44.22 ± 1.11 ^c^	46.77 ± 3.12 ^b^	49.43 ± 0.79 ^a^	50.55 ± 1.64 ^a^	1.32	<0.05
Pro	87.76 ± 0.22 ^c^	88.12 ± 0.19 ^c^	85.54 ± 0.43 ^c^	120.21 ± 4.76 ^a^	90.75 ± 0.84 ^b^	91.87 ± 7.11 ^b^	7.02	<0.05
Lys	127.74 ± 2.47 ^a^	110.08 ± 1.78 ^b^	107.21 ± 5.13 ^b^	130.34 ± 9.05 ^a^	112.42 ± 4.78 ^b^	113.54 ± 5.78 ^b^	9.69	<0.05
Umami taste	298.67 ± 2.79 ^e^	368.42 ± 2.11 ^a^	312.20 ± 1.98 ^d^	329.43 ± 2.09 ^c^	342.62 ± 2.55 ^b^	344.86 ± 2.04 ^b^	4.83	<0.05
Sweet taste	549.59 ± 4.11 ^d^	683.97 ± 4.78 ^a^	587.75 ± 3.85 ^c^	619.36 ± 4.08 ^b^	613.8 ± 3.78 ^b^	619.4 ± 4.26 ^b^	6.37	<0.05
Bitter taste	275.35 ± 1.67 ^c^	274.88 ± 1.79 ^c^	263.83 ± 1.06 ^d^	355.31 ± 1.88 ^a^	289.88 ± 2.01 ^b^	295.48 ± 2.43 ^b^	4.61	<0.05
Acid taste	807.60 ± 3.21 ^d^	847.38 ± 2.79 ^a^	786.85 ± 2.90 ^e^	837.74 ± 3.14 ^b^	822.48 ± 3.29 ^c^	825.84 ± 2.81 ^c^	5.15	<0.05
Sum	1680.97 ± 3.87	1857.15 ± 4.07	1874.98 ± 4.81	2078.86 ± 12.09	1775.17 ± 6.69	1790.85 ± 9.57		

^1^ NC, negative control; PC, Positive control, Samgyetang that was hydrolyzed with flavorzyme before retorting; CME, Samgyetang that was hydrolyzed with *Cordyceps militaris* mushroom extract before retorting; CMB, Samgyetang that was hydrolyzed with *Cordyceps militaris* mushroom extract: bromelain before retorting; CMF, Samgyetang that was hydrolyzed with *Cordyceps militaris* mushroom extract: flavorzyme before retorting; CMBF, Samgyetang that was hydrolyzed with *Cordyceps militaris* mushroom extract: bromelain:flavorzyme before retorting. Umami taste = ∑glutamic acid and aspartic acid; Sweet taste = ∑alanine, glycine, threonine, serine, and proline; Bitter taste = ∑leucine, valine, isoleucine, methionine, and phenylalanine; Acid taste = ∑glutamic acid, aspartic acid, and histidine. SEM, the standard error of the mean. ^a–^^e^. The mean values within the same row with different superscripts are significantly different among the treatments (*p* < 0.05).

**Table 7:** Free amino acid profiles of the broth of Korean ginseng chicken soup that was hydrolyzed with *Cordyceps militaris* mushroom extract.

Variables	Treatments ^1^						SEM	*p*-Value
	NC	PC	CME	CMB	CMF	CMBF		
Asp	32.31 ± 4.13 ^c^	68.01 ± 3.12 ^b^	69.36 ± 1.13 ^b^	73.79 ± 0.87 ^a^	68.83 ± 1.22 ^b^	70.12 ± 3.17 ^a,b^	2.34	<0.05
Glu	180.58 ± 3.75 ^c^	256.19 ± 2.31 ^b^	257.54 ± 2.05 ^b^	261.97 ± 2.89 ^a^	256.98 ± 1.65 ^b^	258.3 ± 1.99 ^a,b^	3.96	<0.05
Ser	74.32 ± 0.77 ^c^	93.10 ± 1.27 ^b^	94.45 ± 3.11 ^b^	98.88 ± 0.08 ^a^	93.89 ± 4.21 ^b^	95.21 ± 2.10 ^a,b^	3.56	<0.05
Gly	42.41 ± 5.08 ^b^	68.11 ± 5.73 ^a^	69.46 ± 4.07 ^a^	73.89 ± 3.70 ^a^	68.90 ± 2.49 ^a^	70.22 ± 2.98 ^a^	4.69	<0.05
Thr	66.22 ± 5.57 ^b^	90.20 ± 1.22 ^a^	91.55 ± 2.05 ^a^	95.98 ± 3.19 ^a^	90.99 ± 4.28 ^a^	92.31 ± 4.82 ^a^	4.41	<0.05
Ala	107.32 ± 7.71 ^b^	256.00 ± 5.12 ^a^	257.35 ± 4.18 ^a^	261.78 ± 4.88 ^a^	256.79 ± 3.64 ^a^	258.11 ± 4.11 ^a^	5.13	<0.05
Val	29.40 ± 1.32 ^d^	52.12 ± 1.29 ^c^	53.47 ± 1.40 ^b,c^	57.93 ± 1.68 ^a^	52.91 ± 0.03 ^c^	54.23 ± 0.19 ^b^	0.20	<0.05
Met	6.21 ± 0.64 ^b^	3.01 ± 0.26 ^c^	4.36 ± 1.74 ^c^	8.79 ± 0.83 ^a^	3.80 ± 1.37 ^c^	5.12 ± 1.68 ^b,c^	0.84	<0.05
Ile	7.56 ± 3.17 ^c^	27.65 ± 0.57 ^b^	29.00 ± 0.11 ^a^	33.43 ± 4.09 ^a^	28.44 ± 4.63 ^a^	29.76 ± 4.11 ^a^	3.77	<0.05
Phe	5.48 ± 3.69 ^c^	26.17 ± 2.09 ^b^	27.52 ± 1.87 ^a,b^	31.95 ± 3.28 ^a^	26.96 ± 1.66 ^b^	28.28 ± 1.04 ^a^	1.87	<0.05
Leu	35.88 ± 2.01 ^c^	55.38 ± 0.09 ^b^	56.73 ± 1.22 ^b^	61.16 ± 2.03 ^a^	56.17 ± 1.60 ^b^	57.49 ± 1.23 ^b^	1.68	<0.05
Arg	47.32 ± 7.11 ^b^	70.21 ± 4.18 ^a^	71.56 ± 3.23 ^a^	75.99 ± 4.04 ^a^	71.00 ± 4.31 ^a^	72.32 ± 2.11 ^a^	4.23	<0.05
His	466.04 ± 2.74 ^a^	456.85 ± 3.16 ^b^	458.20 ± 2.09 ^b^	462.63 ± 1.42 ^b^	457.64 ± 0.98 ^b^	458.96 ± 1.18 ^b^	1.45	<0.05
Tyr	3.09 ± 4.01 ^c^	24.61 ± 2.02 ^b^	25.96 ± 3.27 ^a,b^	30.39 ± 4.92 ^a^	25.40 ± 4.55 ^a,b^	26.72 ± 3.68 ^a,b^	2.01	<0.05
Pro	44.87 ± 3.81 ^b^	66.01 ± 4.02 ^a^	67.36 ± 4.19 ^a^	71.79 ± 4.36 ^a^	66.8 ± 4.23 ^a^	68.12 ± 3.21 ^a^	3.94	<0.05
Lys	84.85 ± 0.09 ^c^	87.97 ± 0.97 ^b^	89.32 ± 1.89 ^b^	93.75 ± 3.05 ^a^	88.76 ± 0.77 ^b^	90.08 ± 2.09 ^a,b^	1.18	<0.05
Umami taste	212.89 ± 1.07 ^c^	324.20 ± 1.24 ^a^	326.90 ± 1.06 ^b^	335.76 ± 1.42 ^a^	325.81 ± 1.33 ^b^	328.42 ± 1.18 ^a^	3.04	<0.05
Sweet taste	335.14 ± 2.13 ^d^	573.42 ± 3.05 ^c^	580.17 ± 2.91 ^b^	602.32 ± 2.89 ^a^	577.37 ± 2.16 ^c^	583.97 ± 2.37 ^b^	6.52	<0.05
Bitter taste	84.53 ± 0.78 ^d^	164.33 ± 0.97 ^c^	171.08 ± 1.03 ^b^	193.26 ± 1.24 ^a^	168.28 ± 0.93 ^b,c^	174.88 ± 1.04 ^b^	3.86	<0.05
Acid taste	678.93 ± 3.12 ^c^	781.05 ± 2.88 ^b^	785.1 ± 3.46 ^b^	798.39 ± 3.07 ^a^	783.45 ± 3.25 ^b^	787.38 ± 2.93 ^b^	6.93	<0.05
Sum	1104.14 ± 7.08	1547.61 ± 6.11	1723.19 ± 5.09	1794.07 ± 6.27	1558.67 ± 6.02	1577.15 ± 4.66		

^1^ NC, negative control; PC, Positive control, Samgyetang that was hydrolyzed with flavorzyme before retorting; CME, Samgyetang that was hydrolyzed with *Cordyceps militaris* mushroom extract before retorting; CMB, Samgyetang that was hydrolyzed with *Cordyceps militaris* mushroom extract: bromelain before retorting; CMF, Samgyetang that was hydrolyzed with *Cordyceps militaris* mushroom extract: flavorzyme before retorting; CMBF, Samgyetang that was hydrolyzed with *Cordyceps militaris* mushroom extract: bromelain:flavorzyme before retorting. Umami taste = ∑glutamic acid and aspartic acid; Sweet taste = ∑alanine, glycine, threonine, serine, and proline; Bitter taste = ∑leucine, valine, isoleucine, methionine, and phenylalanine; Acid taste = ∑glutamic acid, aspartic acid, and histidine.SEM, the standard error of the mean. ^a–^^d^ the mean values within the same row with different superscripts are significantly different among the treatments (*p* < 0.05).

## Data Availability

Data are available within the article.
